# Enhanced Uptake of Iodide from Solutions by Hollow Cu-Based Adsorbents

**DOI:** 10.3390/ma11050769

**Published:** 2018-05-10

**Authors:** Ping Mao, Jinlong Jiang, Yichang Pan, Chuansong Duanmu, Shouwen Chen, Yi Yang, Songlan Zhang, Yonghao Chen

**Affiliations:** 1 Key Laboratory for Palygorskite Science and Applied Technology of Jiangsu Province, Faculty of Chemical Engineering, Huaiyin Institute of Technology, Huaian 223003, China; cduanmu@hyit.edu.cn (C.D.); zhang3266204738@163.com (S.Z.); cyh1161601314@163.com (Y.C.); 2 State Key Laboratory of Materials-Oriented Chemical Engineering, Nanjing University of Technology, Nanjing 210009, China; panyc@njtech.edu.cn; 3 School of Environmental and Biological Engineering, Nanjing University of Science and Technology, Nanjing 210094, China; chensw@njust.edu.cn (S.C.); yangyi@njust.edu.cn (Y.Y.)

**Keywords:** Cu-based adsorbent, hollow structure, iodide, uptake, air

## Abstract

Cu_2_O exhibits excellent adsorption performance for the removal of I^−^ anions from solutions by doping of metallic Ag or Cu. However, the adsorption process only appears on the surface of adsorbents. To further improve the utilization efficiencies of Cu content of adsorbents in the uptake process of I^−^ anions, hollow spheres of metallic Cu, Cu/Cu_2_O composite and pure Cu_2_O were prepared by a facile solvothermal method. Samples were characterized and employed for the uptake of I^−^ anions under various experimental conditions. The results show that Cu content can be tuned by adjusting reaction time. After the core was hollowed out, the uptake capacity of the samples increased sharply, and was proportional to the Cu content. Moreover, the optimal uptake was reached within only few hours. Furthermore, the uptake mechanism is proposed by characterization and analysis of the composites after uptake. Cu-based adsorbents have higher uptake performance when solutions are exposed to air, which further verified the proposed uptake mechanism. Finally, hollow Cu-based adsorbents exhibit excellent selectivity for I^−^ anions in the presence of large concentrations of competitive anions, such as Cl^−^, SO_4_^2−^ and NO_3_^−^, and function well in an acidic or neutral environment. Therefore, this study is expected to promote the development of Cu-based adsorbents into a highly efficient adsorbent for the removal of iodide from solutions.

## 1. Introduction

In many developing countries, including China, rapid growth in demand has given rise to power shortages. However, the major source of energy is still coal, and constitutes about 75% of all energy sources, and the reliance on fossil fuels has generated many air pollutants [1,2]. The World Bank (2007) estimated that the total health cost associated with outdoor air pollution in urban areas of China in 2003 was between 157 and 520 billion China Yuan, accounting for 1.2–3.3% of China’s gross domestic product [2]. Therefore, nuclear power, a kind of green energy, has been a priority of the Chinese government [3]. The Energy Development Strategy Action Plan (2014–2020) noted that China’s operational nuclear installed capacity could reach 58,000 MWe in 2020, with 30,000 MWe more under construction [4]. Unfortunately, as inevitable products of nuclear fission, radioactive iodine species can easily dissolve into solutions. The half-life of radioactive iodine differs from about 8 days (^131^I) to 1.6 × 10^7^ years (^129^I). Radioactive iodine poses a potential toxicity due to its ability to diffuse in solution as an anion and bioaccumulation through the food chain and subsequent dysfunction of the thyroid gland. Therefore, effective uptake of radioactive iodide from solutions will play an irreplaceably vital role in the safety use of nuclear power.

Many means have been used for the treatment of radioactive iodide from contaminated water, such as physical adsorption [5], ion exchange [6], membrane separation [7,8] and chemical precipitation [9], etc. Notably, chemical precipitation is an efficient and easy method that is suitable for safe storage and the emergency treatment of radionuclide in a nuclear dump or polluted water source. Various metal-based adsorbents were reported over the last decades [10,11,12]. Due to their good environmental tolerance, inexpensiveness and relatively low toxicity, Cu^+^-based adsorbents are expected to be the best candidates. Whereas, Cu^2+^ species, oxidized from surface Cu^+^ by dissolved oxygen, appear on the surface of the cuprous adsorbents that hinder the reaction of Cu^+^ and I^−^ anions [13]. Therefore, to address this problem, we doped metallic Ag- or Cu- into Cu_2_O adsorbents [14,15,16]. The adsorption mechanism involves Cu_2_O (Ag_2_O) formed by the reaction of metallic Cu (Ag) and the surface-oxidized CuO adsorbs I^−^ anions. However, the utilization efficiencies of Cu content in these adsorbents are small because of the aforementioned surface adsorption mechanism. Therefore, we want to improve the utilization efficiency of adsorbence through the design of the hollow structure of Cu-based adsorbents.

To the best of our knowledge, the application of various templates, including hard templates, soft templates and self-sacrificial templates, are the main synthetic strategies for the preparation of hollow Cu-based nanomaterials [17,18,19,20,21]. However, it remains a challenge to achieve the facile synthesis of Cu/Cu_2_O composite spheres with a hollow interior.

Herein, we prepared hollow spheres of metallic Cu, Cu/Cu_2_O composite and pure Cu_2_O via a facile solvothermal method. A controllable composition of Cu and Cu_2_O can easily be obtained by adjusting the reaction time. Meanwhile, the hollow Cu-based samples were used to improve its uptake capacity for I^−^ anions from water exposed to air. Furthermore, uptake performances of the hollow Cu, Cu/Cu_2_O and Cu_2_O were studied. Factors affecting the uptake of iodide by the hollow Cu-based samples were also conducted.

## 2. Experimental Section

### 2.1. Synthesis of Hollow Cu-Based Adsorbents

**Figure 1:**
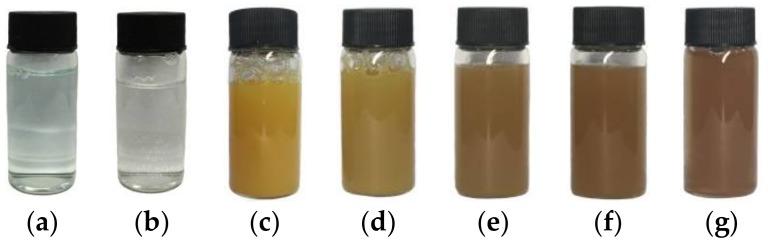
Photographs showing the solution color changes during the course of the as-synthesized samples’ formation process. (**a**) After mixing CuSO_4_·5H_2_O and cetyltrimethylammonium bromide (CTAB); (**b**) immediately after ascorbic acid was added; (**c**) 0 min; (**d**) 5 min; (**e**) 20 min; (**f**) 40 min and (**g**) 60 min after adding NaOH.

All chemicals from commercial sources were of reagent grade and used without further purification. The experiment was derived from one reported in the literature [18]. The synthesis procedure is as follows: 0.4 mmol CuSO_4_·5H_2_O and 20 mmol cetyltrimethylammonium bromide (CTAB) used as soft template for the transcriptive synthesis of hollow nanostructures were dissolved in 200 mL deionized water, followed by vigorous stirring and heating to 80 °C. The color of this mixture was pale blue (Cu^2+^) (Figure 1a). The introduction of 2 mmol of ascorbic acid acted as reducing agent and led to the formation of a colorless transparent solution (Cu^+^) (Figure 1b). The solution was then stirred at 80 °C for 20 min. A yellow precipitate (CuOH) (Figure 1c) was produced when 4 mL of NaOH solution (0.1 M in water) was added dropwise to the above solution. As shown in Figure 1, the color of the solution turned from yellow to brown when the reaction time further increased. After stirring for a certain time, the precipitate was centrifuged, washed sequentially with deionized water and ethanol several times, and then dried at 40 °C for 10 h under vacuum. The as-synthesized samples were designated as S*X*, and the samples after the uptake of I^−^ anions were designated as I-S*X*, where *X* denotes the reaction time (min).

### 2.2. Characterization

The phase purity and crystal structure of the as-synthesized samples were obtained by X-ray diffraction (XRD) on a D8 Advance X-ray diffractometer (Bruker AXS Company, Karlsruhe, Germany) with Cu Kα radiation (A = 1.5406 Å) at 40 KV and 40 mA. The morphologies and microstructures of the samples were examined on an FEI Quanta 250FEG scanning electron microscope (SEM) and an FEI Tecnai G20 transmission electron microscope (TEM) (FEI, Hillsboro, OR, USA). The Brunauer–Emmett–Teller (BET) specific surface area of the samples was measured by using nitrogen adsorption and desorption at a constant temperature of −195 °C on a Micromeritics Tristar 3020 specific surface and porosity analyzer (Micromeritics Instrument Corporation, Norcross, GA, USA). Furthermore, X-ray photoelectron spectroscopy (XPS) spectra of the samples were obtained on a PHI Quantera II electron spectrometer (Physical Electronics GmbH, Ismaning, Germany) using monochromated Al Ka radiation as the excitation source.

### 2.3. Batch Experiments

In this work, all I^−^ anions’ uptake experiments were carried out with non-radioactive ^127^I^−^ (NaI) in aqueous solution because of the high radiation dose and toxicity of radioactive iodide (e.g., ^131^I, ^125^I, ^129^I). Since the concentration of iodide released by nuclear fuel waste is predicted to be below 3 × 10^−4^ M [22], all uptake experiments were carried out with an initial iodide concentration of 10^−4^ M. The as-synthesized samples (50 mg) were added to 50 mL of iodide (NaI) solution in 100 mL Erlenmeyer flasks. The flasks were constantly shaken for 6 h in a shaking incubator at 150 rpm. Unless otherwise mentioned, all experiments with solutions exposed to air were tested. Afterward, the solids and solutions were separated by centrifugation, and the supernatants were analyzed by an ultraviolet spectrophotometer (UV–vis) at 227 nm to determine the remaining I^−^ anions in the solutions. The effects of the uptake parameters (initial iodide concentration, solution pH, uptake time, etc.) were investigated. The initial iodide concentrations ranged from 0.04 to 0.40 mM. Kinetic studies were conducted using an iodide concentration of 0.40 mM for different time intervals. The pH of the solution was adjusted to values between 3 and 10 using 0.1 M HCl or 0.1 M NaOH. The selective uptake studies were determined using iodide concentration of 0.40 mM in the presence of high concentrations of Cl^−^, NO_3_^−^, SO_4_^2−^, and CO_3_^2−^ anions. The iodide concentration was measured by iodine blue spectrophotometry. The uptake performances of the hollow Cu-based adsorbents in an Ar_2_ or O_2_ environment were further studied. Every experiment was analyzed in triplicate, and the average values were utilized to calculate the uptake capacities. The uptake capacity (*q_e_*), uptake efficiency (*η*) and uptake rate (*r*) were calculated using the following mathematical equations, respectively:

(1) $$q_{e}=\left(C_{0}-C_{e}\right)\frac{V}{m}$$

(2) $$\eta =\frac{C_{0}-C_{e}}{C_{0}}\times 100\%$$

(3) $$r=\frac{q_{e}}{t_{e}}$$

where *q_e_* is the amount of iodide uptake on the as-synthesized samples at equilibrium time (mmol g^−1^), *C*_0_ and *C_e_* are the initial and equilibrium iodide concentration (mM) in the solution, respectively, *V* is the volume (L) of iodide solution, and *m* is the mass of the as-synthesized samples (g), *t_e_* is equilibration time.

## 3. Results and Discussion

### 3.1. Characterization

**Figure 2:**
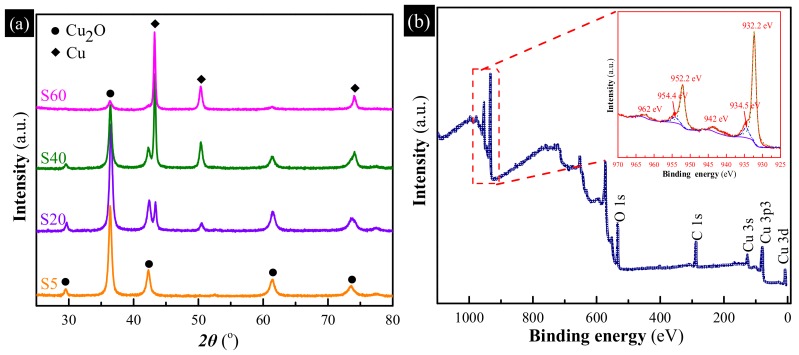
(**a**) X-ray diffraction (XRD) patterns of the as-synthesized samples obtained with different reaction time, inset are photos of the as-synthesized samples; (**b**) X-ray photoelectron spectroscopy (XPS) patterns of S40, inset is the high-resolution spectrum of the Cu 2p peaks.

XRD measurements were used to gain insights into the chemical composition of the as-synthesized samples, as shown in Figure 2a. It can be observed that S5 exhibited many diffraction peaks at 29.6°, 36.5°, 42.4°, 61.5° and 73.7°, corresponding to the diffractions from the (110), (111), (200), (220) and (311) crystalline planes of Cu_2_O (JCPDS 65-3288) [23]. By increasing the reaction time, the characteristic peaks of Cu_2_O become weaker; however, the peaks at 43.3°, 50.4° and 74.1°, indexed to the (111), (200) and (220) crystalline planes of metallic Cu (JCPDS 65-9026) [24], become stronger, revealing the generation of an increasingly metallic Cu with the reaction time increased. Moreover, the metallic Cu content of four samples, calculated according to the peak areas of XRD patterns, is about 0, 24.2%, 60.6%, and 100%, respectively. Meanwhile, inset in Figure 2 shows the photos of the as-synthesized samples. The color gradually turns from yellow into green and then brown, with the changes resulting from the growth of the content of metallic Cu. Moreover, the surface properties of S40 were also examined by XPS measurement. As shown in Figure 2b, the binding energies are calibrated by C 1s (284.8 eV), Cu 2p peaks were investigated in detail to obtain the chemical state and structural characteristics. The main peaks at 932 eV and 952 eV of the Cu 2p XPS spectrum (inset in Figure 2b) can be fitted to two peaks, respectively. The peaks at 932.2 eV and 952.2 eV are attributed to the binding energies of Cu 2p_3/2_ and Cu 2p_1/2_ of Cu_2_O, respectively [25]. The peaks at 934.5 eV and 954.4 eV and two small satellite peaks at 942 eV and 962 eV, corresponding to the binding energies of CuO [26], confirm the presence of CuO on the surface of S40. No CuO phase can be observed from XRD patterns since XPS is much more sensitive compared to XRD.

**Figure 3:**
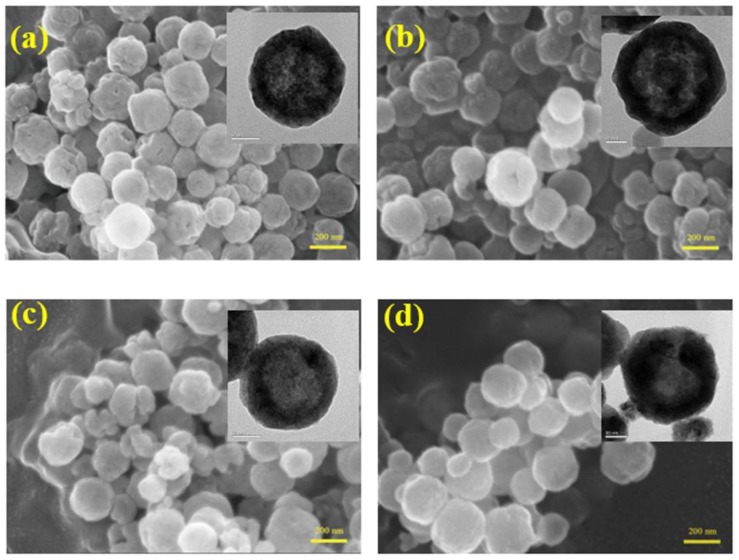
Scanning electron microscope (SEM) and transmission electron microscope (TEM) (inset) photos of S5 (**a**); S20 (**b**); S40 (**c**) and S60 (**d**).

Figure 3 displays SEM and TEM images of the as-synthesized samples. As revealed in the SEM images, all as-synthesized samples possessed a spherical and subglobular shape with an average size of 200 nm that consisted of many nanoparticles. Lots of pores existed on the surface of the samples. However, the number of the pores decreased with increasing reaction time. The BET results further confirmed this point. The BET surface areas of S5, S20, S40 and S60, as measured by the nitrogen adsorption–desorption method, was about 5.13, 5.01, 3.53 and 1.71 m^2^ g^−1^, respectively. Meanwhile, TEM images (inset in Figure 3) showed that the interior space of the samples is empty, and all as-synthesized samples are hollow spheres. TEM images further verified that the samples consisted of many nanoparticles, which are consistent with the SEM results. The shells of these hollow spheres are single-crystalline; this property makes these structures more stable [18].

### 3.2. Uptake Performance Studies

#### 3.2.1. Uptake Equilibrium Isotherms

**Figure 4:**
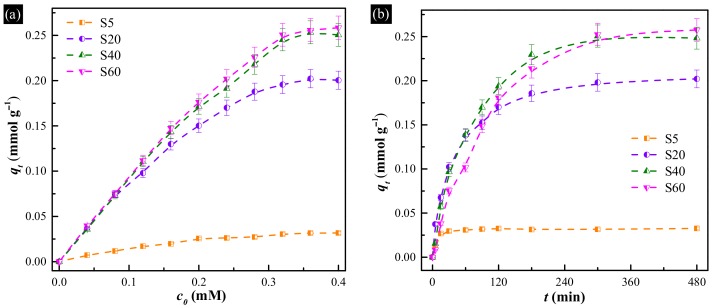
(**a**) Uptake isotherms of all hollow Cu-based adsorbents for I^−^ anions over 12 h; (**b**) uptake kinetics of all hollow Cu-based adsorbents in 0.4 mM I^−^ anions.

To understand the uptake performance of the hollow Cu-based adsorbents, the I^−^ anions equilibrium uptake isotherms of all synthesized samples with different initial iodide concentrations were obtained. The results are shown in Figure 4a. It is evident that the uptake capacities of all samples progressively increase with increasing concentration of I^−^ anions, and finally reach the saturation states. The uptake efficiencies of S40 and S60 for I^−^ anions are higher than 99% when the concentration of I^−^ anions in under 0.16 M. Moreover, the maximum uptake capacity of all samples increases as the content of metallic Cu increased, which is similar to the previous report [15]. It is also apparent that the quantity of I^−^ anions adsorbed per unit mass is 0.03 mmol g^−1^ for S5, 0.20 mmol g^−1^ for S20, 0.25 mmol g^−1^ for S40 and 0.26 mmol g^−1^ for S60, respectively. Furthermore, as summarized in Table 1, by comparing the uptake capacity of all hollow Cu-based adsorbents with the previous reported Cu-based adsorbents, it could be found that the uptake capacity of pure Cu_2_O increased from 0.02 to 0.03 mmol g^−1^. Meanwhile, the content of metallic Cu declined by 7.6%, however, the uptake capacity increased from 0.18 mmol g^−1^ for Cu/Cu_2_O hybrid to 0.03 mmol g^−1^ for S20. This implies that the samples with hollow structure have higher utilization efficiency of Cu.

#### 3.2.2. Uptake Kinetics

The Langmuir and Freundlich isotherm models were used for supplementary investigations into the uptake process. The mathematical representations of them are described as Equations (4) and (5), respectively:

(4) $$\frac{C_{e}}{q_{e}}=\frac{1}{q_{m}b}+\frac{C_{e}}{q_{m}}$$

(5) $$\ln q_{e}=\frac{1}{n}\ln C_{e}+\ln K_{f}$$

where *q_m_* (mmol g^−1^) is the theoretical maximum uptake capacity, *b* and *K_f_* are the uptake constants of the Langmuir and Freundlich models, respectively, and *n* is the Freundlich linearity index.

The Langmuir adsorption model assumes that adsorption occurs on a homogeneous surface by monolayer adsorption without any interactions between the adsorbed anions [30,31]. As depicted in Figure S1a and Table S1, the good straight line shows that the uptake process followed the Langmuir isotherm. The monolayer uptake capacity was estimated to be 0.05, 0.22, 0.29 and 0.27 mmol g^−1^ for S5, S20, S40 and S60, respectively, which is similar to the above uptake isotherm results. By contrast, the Freundlich isotherm is an empirical model and can be used to describe adsorption on heterogeneous surfaces as well as multilayer adsorption [32]. As shown in Figure S1b and Table S1, the linear regressions of all samples did not fit well, compared with those from the Langmuir model. This further confirms that the Langmuir adsorption model is a good model of the adsorption system, which suggests the homogeneous nature of uptake sites on the surface of the hollow Cu-based samples with a monolayer adsorption.

To determine the uptake rates of the hollow Cu-based adsorbents, the kinetic of the uptake of I^−^ anions was plotted at initial iodide concentration of 0.40 mM, as shown in Figure 4b. The result revealed that the uptake reached optimal removal within 60, 300, 300 and 480 min for S5, S20, S40 and S60, respectively. After reaching the saturation value, a continuous and smooth graph was obtained. Therefore, the uptake rates (*r*) of all samples were 0.03, 0.04, 0.05 and 0.03 mmol g^−1^ h^−1^, which indicates that S40 is the best candidate for the removal of I^−^ anions.

The pseudo-first-order and pseudo-second-order models [33] were used to perform data fitting. The mathematical representations are given below:

(6) $$\ln\left(q_{m}-q_{t}\right)=\ln q_{m}-k_{1}t$$

(7) $$\frac{t}{q_{t}}=\frac{1}{k_{2}q_{m}^{2}}+\frac{1}{q_{m}}t$$

where *q_t_* (mmol g^−1^) represents the uptake capacity at time *t* (min), *k*_1_ and *k*_2_ (g mmol^−1^ min^−1^) are the pseudo-first-order rate constant and the pseudo-second-order rate constant. As depicted in Figure S2, the graphs of *t*/*q_t_* versus *t* and ln(*q_e_* − *q_t_*) versus *t* were plotted. The pseudo-first-order rate constants and pseudo-second-order rate constants are summarized in Table S2. According to the linear regression coefficients (R^2^) of the kinetic models, the second-order kinetic of every sample fits better than its first-order kinetic during the uptake process. The pseudo-second-order kinetic model is used to predict the kinetic behavior of adsorption with chemical adsorption being the rate-controlling step [33]. Therefore, chemical adsorption dominated the uptake process of the hollow Cu-based adsorbents.

### 3.3. Uptake Mechanism

**Figure 5:**
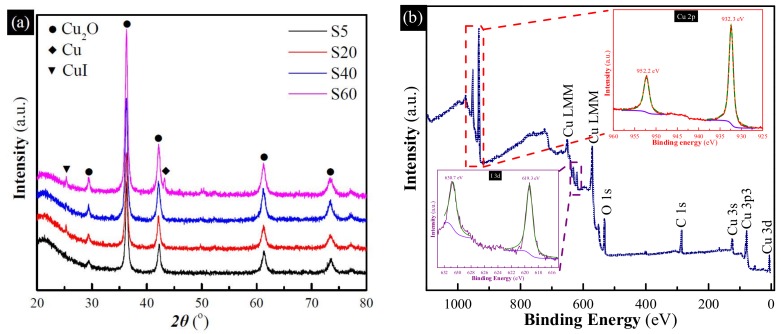
(**a**) XRD patterns of hollow Cu-based adsorbents after the uptake of I^−^ anions; (**b**) XPS patterns of S40 after the uptake of I^−^ anions, inset are the high-resolution spectra of Cu 2p and I 3d peaks, respectively.

To analyze the uptake mechanism of the hollow Cu-based adsorbents, XRD and XPS measurement of the samples after the uptake of I^−^ anions were conducted. As shown in Figure 5a, the diffraction peaks assigned to metallic Cu become weaken, but a new peak ascribed to CuI (JCPDS 06-0246) [34] at 25.5° can be found. Meanwhile, Figure S3 shows that the solution colors of all hollow Cu-based adsorbents change to yellow or dark yellow. This further implied that the content of metallic Cu decreased. All of this implied that the CuI and metallic Cu were the uptake product and reactant in the uptake process, respectively. Moreover, XPS patterns of I-S40 (Figure 5b) revealed that the peaks ascribed to Cu^2+^ disappeared, indicating that Cu^2+^ is also the reactant. Furthermore, the I 3d core level appeared at the binding energies of around 630.7 eV and 619.3 eV assigned to CuI [35], further confirming that CuI was the uptake product. Therefore, the probable uptake mechanism for hollow Cu-based adsorbents can be proposed. Pure Cu_2_O hardly reacts with I^−^ anions because of the CuO surface layer. For the uptake mechanism of Cu/Cu_2_O and pure metallic Cu, part metallic Cu and Cu_2_O can be oxidized to CuO by dissolved oxygen. Then, metallic Cu reacts with CuO to generate Cu_2_O. Subsequently, generated Cu_2_O reacts with I^−^ anions to form CuI. In brief, the uptake processes can be summarized as follows:

(8) $$\mathrm{Cu}+\mathrm{CuO}↔{\mathrm{Cu}}_{2}O$$

(9) $${\mathrm{Cu}}_{2}O+2I^{-}+H_{2}O\to 2\mathrm{CuI}+2{\mathrm{OH}}^{-}$$

**Figure 6:**
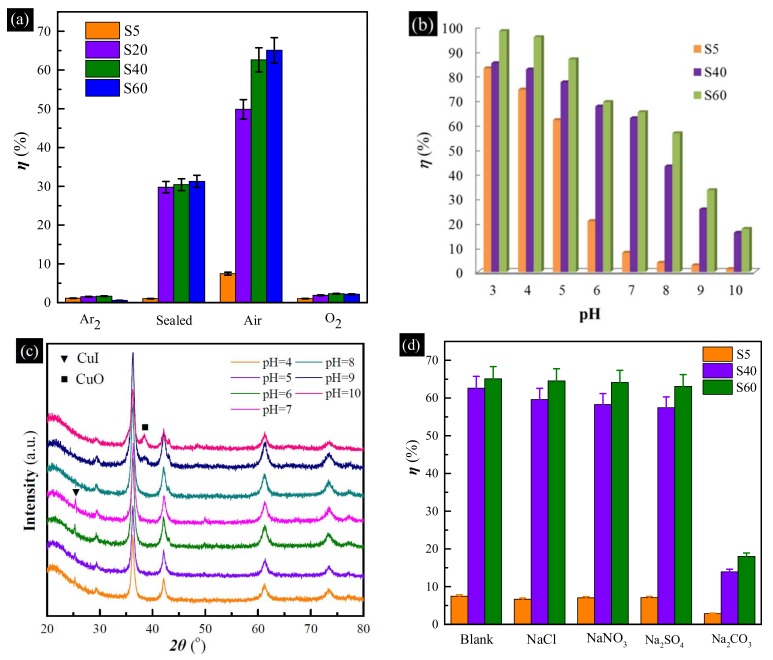
(**a**) Effect of different atmospheres on the uptake of I^−^ anions by hollow Cu-based adsorbents; (**b**) uptake efficiency of I^−^ anions in the pH range from 3 to 10; (**c**) XRD patterns of S40 after the uptake of I^−^ anions at different pH values; (**d**) effect of high competitive anions on the uptake of I^−^ anions by hollow Cu-based adsorbents.

To further verify the importance of the dissolved oxygen during the uptake process of the Cu-based adsorbents, the uptake experiments were performed in Ar_2_, O_2_ and sealed air atmosphere, respectively. The results are presented in Figure 6a. The uptake efficiency of every Cu-based adsorbent decreased sharply in the air atmosphere. Even when the flask was sealed, the uptake efficiencies of all hollow Cu-based adsorbents decreased to about 1.0%, 29.8%, 30.4% and 31.3%, respectively. This further verified that oxygen is of importance in the uptake. However, the uptake efficiencies of the samples also decreased sharply in the O_2_ atmosphere. This indicates that excess oxygen is detrimental to the uptake. The probable reason is that metallic and Cu_2_O can be oxidized to CuO by excess oxygen, or the uptake reactions have been severely disrupted by the floating gas.

### 3.4. Effect of Factors

#### 3.4.1. Effect of pH

To investigate the effect of pH on the uptake of I^−^ anions by hollow Cu-based adsorbents, pH values ranging from 3 to 10 and the samples of S5, S40 and S60 were chosen. As shown in Figure 6b, with the increase in pH of the solution, the uptake efficiency is found to decrease in the case of all samples, implying that acidic medium is helpful for the uptake of I^−^ anions by the hollow Cu-based adsorbents, but the alkaline environment has an adverse effect. XRD patterns (Figure 6c) of S40 after the uptake of I^−^ anions at different pH values can be used to explain the above phenomenon. When pH value decreased from 7 to 3, a diffraction peak at 25.5°, corresponding to the diffraction from the (111) crystalline plane of CuI (JCPDS 06-0246) [34], can be found in the pH range of 3–7. CuO, Cu_2_O and Cu can partially be dissolved in an acidic solution, forming Cu^2+^ and Cu^+^ species, which are helpful for generating CuI precipitate [29]. However, another diffraction peak at 38.7°, assigned to the (111) crystalline plane of CuO (JCPDS 48-1548) [36] became stronger when pH value increased to 9. In an alkaline environment, Cu and Cu_2_O can easily be oxidized to CuO that cannot reaction with I^−^ anions [15].

#### 3.4.2. Selective Uptake

To understand the selective uptake of I^−^ anions by the hollow Cu-based adsorbents, a series of experiments in the presence of high concentrations of Cl^−^, NO_3_^−^, SO_4_^2−^ and CO_3_^2−^ anions (40 mM) were conducted. The uptake results are shown in Figure 6d. No obvious difference can be found in the presence of high concentrations of Cl^−^, NO_3_^−^ and SO_4_^2−^ anions. However, uptake performance of all samples drops off rapidly in the presence of high concentrations of CO_3_^2−^ anions. It is evident from Figure S4 that many diffraction peaks assigned to CuI can be found in the presence of Cl^−^, NO_3_^−^ and SO_4_^2−^ anions, and the results were in accordance with those obtained without the competitive anions. However, many peaks, corresponding to CuO, can be found in the presence of CO_3_^2−^ anions. Cu and Cu_2_O can also be oxidized to CuO in a weakly alkaline environment. As we know, both CuI and CuCl are precipitates. However, the solubility products (*K_sp_^Φ^*) of CuCl and CuI are 1.2 × 10^−6^ and 1.1 × 10^−12^, respectively. When the concentration of Cl^−^ is about 40 mM, in order to ensure the generation of CuCl precipitate, the minimum concentration of Cu^+^ is 3 × 10^−^^5^ M according to *K_sp_^Φ^*(CuCl) = *c*(Cu^+^) × *c*(Cl^−^),. Moreover, the residual concentration of I^−^ is 3.67 × 10^−^^8^ M according to *K_sp_^Φ^*(CuI) = *c*(Cu^+^) × *c*(I^−^). Then when the initial concentration is 0.4 mM, the uptake efficiency can reach about 99.0825%. Therefore, Cl^−^ cannot affect the iodide uptake of Cu-based adsorbents.

## 4. Conclusions

Hollow spheres of metallic Cu, Cu/Cu_2_O composite and pure Cu_2_O were prepared by a facile solvothermal method. XRD analyses revealed that the Cu content of all samples can be tailored by the reaction time. TEM and SEM measurements showed that all hollow samples consisted of nanoparticles and the pores on the surface were gradually disappearing with increasing Cu content. CuO was appearing on the surface of the samples according to measurement by XPS analyses. Comparing the uptake capacity of solid and hollow Cu-based adsorbents, as-synthesized hollow samples have higher utilization efficiencies of Cu content. Meanwhile, all Cu-based adsorbents obtain the highest uptake performance when solutions are exposed to air. The uptake capacity of hollow Cu-based adsorbents increased with increasing doping content of metallic Cu. The maximum adsorption capacity of pure metallic Cu is 0.26 mmol g^−1^. Moreover, the uptake reaches optimal removal within only a few hours. Furthermore, the uptake mechanism is proposed and verified by characterization of the composites after the uptake and analysis of the experiments. Finally, the hollow Cu-based adsorbents exhibit excellent selectivity for I^−^ anions in the presence of large concentrations of competitive anions, such as Cl^−^, SO_4_^2−^ and NO_3_^−^, and function well in the acidic or neutral environments. Given the merits of this adsorbent, the hollow Cu-based adsorbents may be a more effective candidate for the uptake of I^−^ anions from water in practical applications

## Figures and Tables

**Table 1:** Comparison of the iodide uptake capacities of several previously reported Cu-based adsorbents.

Sample	pH Value	Temperature (°C)	Removal Capacity (mmol g^−1^)	Ref.
S5 (Cu_2_O)	7	25	0.03	This work
S20 (24.2%-Cu/Cu_2_O)	7	25	0.20	This work
S40 (60.6%-Cu/Cu_2_O)	7	25	0.25	This work
S60 (Cu)	7	25	0.26	This work
Cu_2_O *^a^*	6.7	25	0.002	Ref. [13]
31.8%-Cu/Cu_2_O	7	25	0.18	Ref. [14,15]
Cu_2_O	7	25	0.02	Ref. [14,15]
1.0%-Ag@Cu_2_O	7	25	0.20	Ref. [14]
Cu	7	22	0.05	Ref. [27]
CuO	7	22	0.002	Ref. [27]
Cu_2_O	7	22	0.016	Ref. [27]
Cu_2_S *^a^*	7	25	0.048	Ref. [28]
CuCl *^b^*	7	25	2.0	Ref. [29]

*^a^* Experiments were carried out in 0.1 M NaClO_4_; *^b^* Experiments were carried out in 130 mg L^−1^ Na_2_SO_3_.

## Supplementary Materials

The following are available online at http://www.mdpi.com/1996-1944/11/5/769/s1. Figure S1. Fitting curves of the Langmuir (a) and Freundlich models (b) for the uptake isotherms of all hollow Cu-based adsorbents. Figure S2. Pseudo-first-order (a) and pseudo-second-order (b) kinetics models for iodide anions removal on all as-synthesized samples. Figure S3. Photographs showing the solution color changes during the uptake process of all Cu-based adsorbents. (a) o h, (b) 1 h. (c) 1.5 h, (d) 4.5 h, (e) 6 h and (f) 12 h. Figure S4. XRD patterns of S5, S40 and S60 after the uptake of I^−^ anions in the presence of high concentrations of Cl^−^ (a), NO_3_^−^ (b), SO_4_^2−^ (c), and CO_3_^2−^ (d) anions. Table S1. Isotherm parameters for the uptake of I^−^ anions by all hollow Cu-based adsorbents. Table S2. Kinetic parameters for the uptake of I^−^ anions by all hollow Cu-based adsorbents.
