# Supplementary material for: Enhanced Uptake of Iodide from Solutions by Hollow Cu-Based Adsorbents
Source: Materials (Basel). 2018 May 10;11(5):769. doi: 10.3390/ma11050769 (PMC5978146; doi:10.3390/ma11050769)
Supplement: Supplementary file 1 [file materials-11-00769-s001.pdf]

Enhanced Uptake of Iodide from Solutions by Hollow Cu-Based Adsorbents

Ping Mao [1,*], Jinlong Jiang [1,2,*], Yichang Pan [2], Chuansong Duanmu [1], Shouwen Chen [3], Yi Yang [3], Songlan Zhang [1] and Yonghao Chen [1]

1. Key Laboratory for Palygorskite Science and Applied Technology of Jiangsu Province, Faculty of Chemical Engineering, Huaiyin Institute of Technology, Huaian 223003, China; cduanmu@hyit.edu.cn (C.D.); zhang3266204738@163.com (S.Z.); cyh1161601314@163.com (Y.C)
2. State Key Laboratory of Materials-Oriented Chemical Engineering, Nanjing University of Technology, Nanjing 210009, China; panyc@njtech.edu.cn
3. School of Environmental and Biological Engineering, Nanjing University of Science and Technology, Nanjing 210094, China; chensw@njust.edu.cn (S.C.); yangyi@njust.edu.cn (Y.Y.)

* Correspondence: pingmao@hyit.edu.cn (P.M.); jiangjinlong75@163.com (J.J.); Tel.: +86-517-8355-9056 (P.M.)

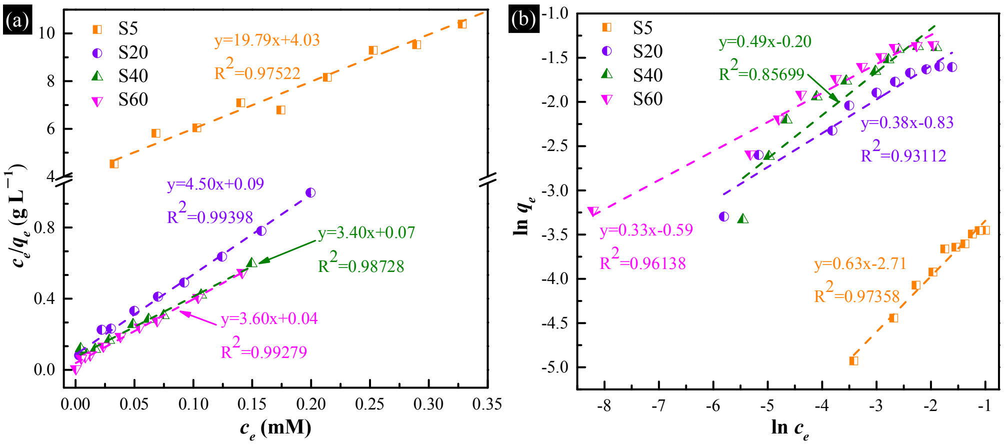

Figure S1. Fitting curves of the Langmuir (a) and Freundlich models (b) for the uptake isotherms of all hollow Cu-based adsorbents.

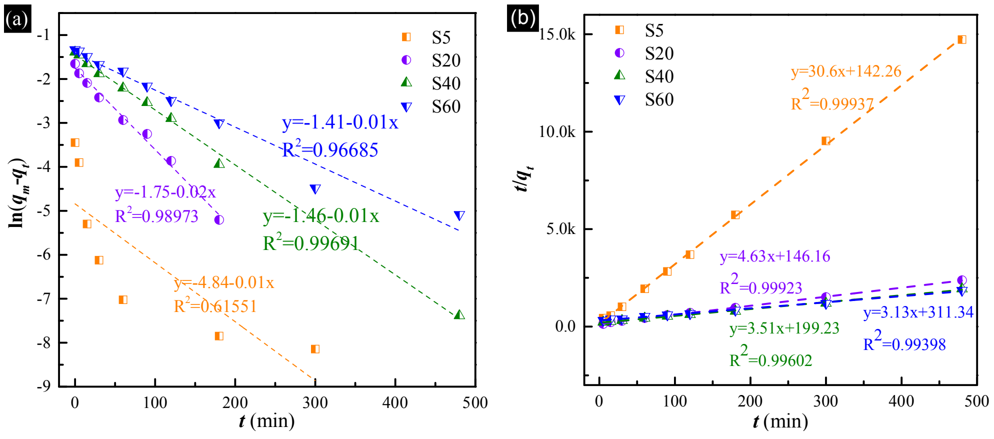

Figure S2. Pseudo-first-order (a) and pseudo-second-order (b) kinetics models for iodide anions removal on all as-synthesized samples.

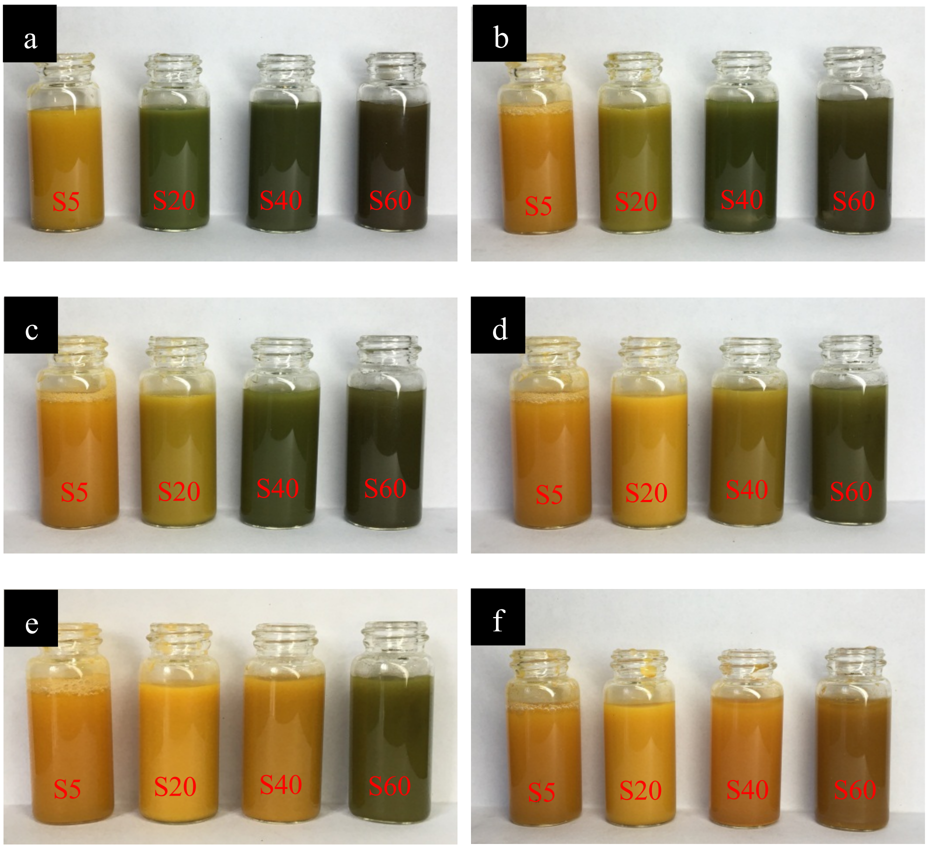

Figure S3. Photographs showing the solution color changes during the uptake process of all Cu-based adsorbents. (a) o h, (b) 1 h. (c) 1.5 h, (d) 4.5 h, (e) 6 h and (f) 12 h.

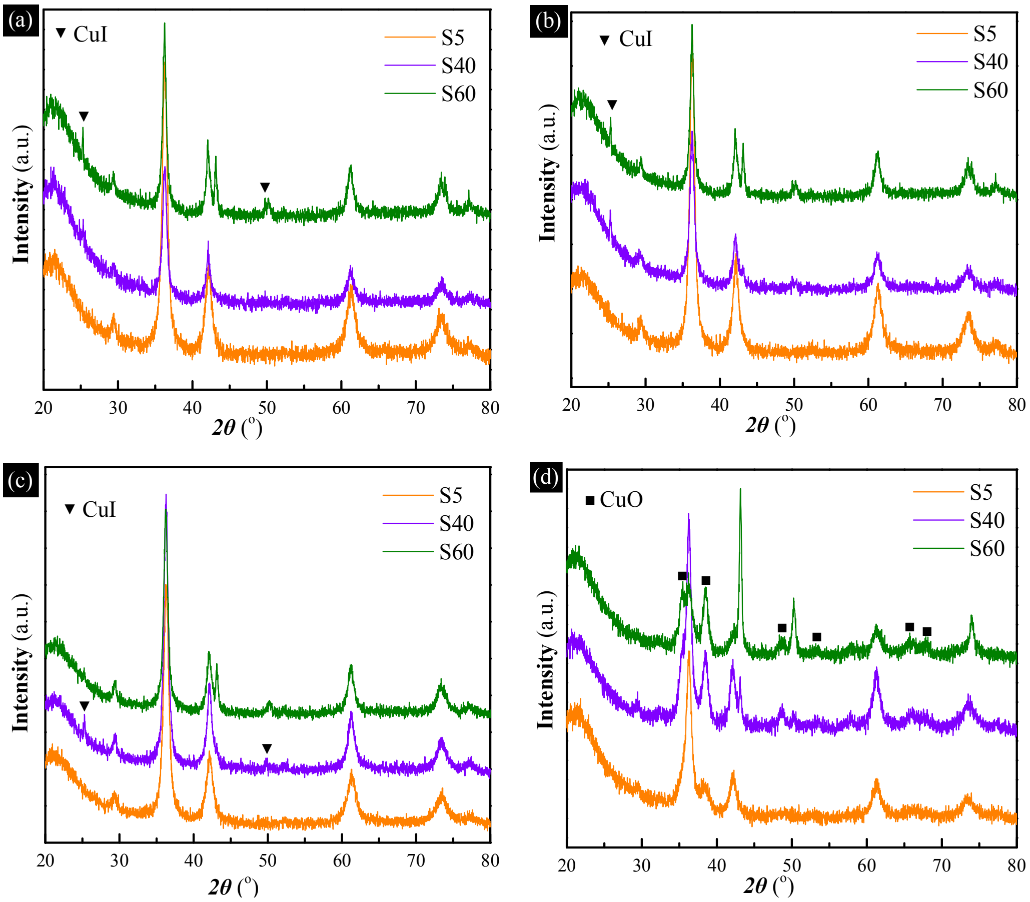

Figure S4. XRD patterns of S5, S40 and S60 after the uptake of I^- anions in the presence of high concentrations of Cl^- (a), NO_3^- (b), SO_4^{2-} (c), and CO_3^{2-} (d) anions.

Table S1 Isotherm parameters for the uptake of I^- anions by all hollow Cu-based adsorbents.

**Sample**	**Langmuir model**			**Frenudlich model**		
	q_m	b	R^2	K_f	$1/n$	R^2
S5	0.05	0.01	0.97522	0.07	0.63	0.97358
S20	0.22	2.47	0.99398	0.44	0.38	0.93112
S40	0.29	4.20	0.98728	0.82	0.49	0.85699
S60	0.27	6.94	0.99279	0.56	0.33	0.96138

Table S2 Kinetic parameters for the uptake of I^- anions by all hollow Cu-based adsorbents.

**Adsorbent**	Pseudo-first-order			Pseudo-second-order		
	k_1 (g mmol^{-1} min^{-1})	q_m (mmol^{-1})	R^2	k_2 (g mmol^{-1} min^{-1})	q_m (mmol^{-1})	R^2
S5	0.01	0.01	0.61551	6.58	0.03	0.99937
S20	0.02	0.17	0.98973	0.15	0.22	0.99923
S40	0.01	0.23	0.99691	0.06	0.29	0.99602
S60	0.01	0.24	0.96685	0.03	0.31	0.99398
